# Leprosy

**Published:** 1901-12-07

**Authors:** 


### LEPROSY.

The announcement that Mr. Jonathan Hutchinson is proceeding to South Africa with the object of studying leprosy, draws attention afresh to that ancient disease, and also to the particular view as to its causation, of which Mr. Hutchinson has for so long been the chief exponent.

Of the nature of leprosy, bacteriology has taught us somewhat. Of its causation we know nothing beyond the somewhat bald statement that it is endemic in certain districts. Although its essential cause is a microbe, and although it must therefore be classed amongst the infective disorders, the evidences of contagion are of the slightest. Within endemic areas cases are met with in which direct infection seems to have occurred, but in non-leprous districts the imported disease (for it often is imported) shows no tendency to spread. Now and again, however, leprosy spreads into new districts, so that, while the disease is waning in some parts of the world, new areas are being added to its kingdom in other directions; and we may well believe that one reason at least which makes South Africa a place of interest to Mr. Hutchinson is the behaviour of leprosy in that region during comparatively recent years. Mr. Hutchinson is a strong supporter of the theory that the origin and causation of leprosy is connected with the custom of eating uncooked fish. The idea that fish-eating has something to do with leprosy dates back from long ago; in fact, it is one which almost naturally arises in the minds of those who observe the geographical distribution of the disease and note how it tends to cling to the coast line or to the borders of great rivers, where people live largely upon fish. Many facts have, however, been brought up against the fish theory; especially has it been pointed out that leprosy also prevails in certain places far from rivers or seas, where fish cannot be obtained, as, for example, in the mountains of Kurdistan, and in many parts of India among castes which religiously abstain from all animal food, including fish. To this, however, Mr. Hutchinson answers that in India, where a monotonous rice diet prevails, condiments are largely used, and that a not uncommon constituent of such condiments is fish in an uncooked and partially decomposed condition. He also shows how far inland salted and dried fish may be carried along trade routes.

The great blow to the fish theory came, of course, with the discovery of the leprosy bacillus. This, however, has not discouraged Mr. Hutchinson, and he certainly brings forward a long array of arguments in favour of some causal relation existing between the consumption of imperfectly cooked fish and the occurrence of leprosy. He holds, and indeed shows, that leprosy has nothing whatever to do with race or climate; that it has no necessary connection with poverty or personal neglect; that it prevails extensively in some places in which as regards food, clothing, and climate, no sort of hardship exists; that it certainly is not contagious in the ordinary sense of the word, and yet that its infective cause (if, as its bacteriology suggests, we are to regard it as an infective disease) must be one which acts with considerable intensity when the conditions are appropriate, seeing how comparatively short a residence in an endemic area may give rise to the disease. The legitimate inference of all this is, Mr. Hutchinson says, that the disease arises from some dietetic habit, and that "as gout is yielding before tea, coffee, and temperance, so will leprosy yield before the substitution of cereals, potatoes, and butcher's meat for salted fish." The relation of leprosy to South Africa is one of great interest. When the Dutch founded Capetown, there was, he says, no leprosy amongst the inland natives. A century later, two Dutch farmers near to Capetown were found to be lepers, and since then the disease has been steadily increasing and spreading northwards amongst both native and European races. Its chief foci, however, are still in and near to Capetown and other coast towns. Why then has this non-contagious disease increased and spread up the country? and why does it still have Capetown as its main focus of distribution? To this Mr. Hutchinson answers by saying that the disease began amongst the Dutch near Capetown after the establishment of a fish-curing factory at that place. It spread amongst the Hottentot slaves and others after salted fish had become an ordinary article of diet in the daily ration. It increases because, with the development of commerce, the supply of salt fish inland has become more and more common; and, finally, Capetown is the principal focus because the largest salt-fish factory is situated there. According to this view one may say that what is endemic in leprosy areas is not so much leprosy as the social custom of eating uncooked fish; much in the same way as trichina disease may be apparently endemic in certain countries in which what really is endemic is the habit of eating uncooked pork. There is much in the fish theory, and in the striking way in which Mr. Hutchinson has from time to time enforced it, which is very attractive. But it is to be confessed that the bacteriology of the disease stands much in the way of its acceptance.

